# Regulation of influenza A virus infection by Lnc‐PINK1‐2:5

**DOI:** 10.1111/jcmm.17249

**Published:** 2022-02-24

**Authors:** Samuel Pushparaj, Zhengyu Zhu, Chaoqun Huang, Sunil More, Yurong Liang, Kong Lin, Kishore Vaddadi, Lin Liu

**Affiliations:** ^1^ Oklahoma Center for Respiratory and Infectious Diseases Oklahoma State University Stillwater Oklahoma USA; ^2^ The Lundberg‐Kienlen Lung Biology and Toxicology Laboratory Department of Physiological Sciences Oklahoma State University Stillwater Oklahoma USA

**Keywords:** antiviral, host factors, influenza virus, Lnc‐PINK1‐2, long non‐coding RNAs, TXNIP

## Abstract

Influenza virus causes approximately 291,000 to 646,000 human deaths worldwide annually. It is also a disease of zoonotic importance, affecting animals such as pigs, horses, and birds. Even though vaccination is being used to prevent influenza virus infection, there are limited options available to treat the disease. Long noncoding RNAs (lncRNAs) are RNA molecules with more than 200 nucleotides that do not translate into proteins. They play important roles in the physiological and pathological processes. In this study, we identified a novel transcript, Lnc‐PINK1‐2:5 that was upregulated by influenza virus. This lncRNA was predominantly located in the nucleus and was not affected by type I interferons. Overexpression of Lnc‐PINK1‐2:5 reduced the influenza viral mRNA and protein levels in cells as well as titres in culture media. Knockdown of Lnc‐PINK1‐2:5 using CRISPR interference enhanced the virus replication. Antiviral activity of Lnc‐PINK1‐2:5 was independent of influenza virus strains. RNA sequencing analysis revealed that Lnc‐PINK1‐2:5 upregulated thioredoxin interacting protein (TXNIP) during influenza virus infection. Overexpression of TXNIP reduced influenza virus infection, suggesting that TXNIP is an antiviral gene. Knockdown of TXNIP abolished the Lnc‐PINK1‐2:5‐mediated increase in influenza virus infection. In conclusion, the newly identified Lnc‐PINK1‐2:5 isoform is an anti‐influenza lncRNA acting through the upregulation of TXNIP gene expression.

**Importance**

Influenza virus remains as the major respiratory pathogen that causes a significant public health burden worldwide. There is a constant need to develop new antivirals due to incomplete protection of vaccinations as well as virus assortment and re‐emergence. Several host long noncoding RNAs (lncRNAs) have been shown to have critical functional roles in influenza virus replication. In this study, we identified the new transcript, named as Lnc‐PINK1‐2:5 that is the major transcript in lung epithelial cells. Lnc‐PINK1‐2:5 was induced during influenza virus infection and was regulated through c‐Myc signalling pathway. Lnc‐PINK1‐2:5 reduced influenza virus replication via the upregulation of thioredoxin interacting protein (TXNIP). This study thus highlights the importance of identifying new lncRNAs that could be potential therapeutic targets in the future.

## 1 INTRODUCTION

Influenza viruses are globally important and recognized respiratory pathogens. It belongs to the *Orthomyxoviridae* family. Influenza type A, B, C, and D are the members of this family.^1^, ^2^ Influenza A and B viruses cocirculate in humans and cause seasonal epidemics and occasional pandemics. It causes an acute and highly contagious respiratory illness that commonly affects the upper respiratory tract. However, sometimes the virus causes primary pneumonia by affecting the lower respiratory tract. Severe infections are observed in very young or elderly age groups or in individuals suffering from chronic diseases.^3^ Even though vaccination is available, seasonal influenza infection causes 291,000 to 646,000 human deaths every year worldwide.^4^ Also, the current drugs treating influenza infection are gaining significant resistance.^5^ The unpredictability of the influenza virus is due to the antigenic shift and drift, which cause reassortment and mutations in the viral surface proteins. This paves the way for the zoonotic capability of the influenza A virus (IAV), leading to the evolution of antigenically novel viral strains that can efficiently replicate in humans. Being such a disease of high pathogenicity and the ability to spread, it is crucial to understand the regulation of influenza virus infection in order to formulate a control strategy.

Only 2% of the human genome encodes for proteins. However, approximately 70% of them are transcribed into RNAs. Based on the size, the protein‐noncoding RNAs (ncRNAs) are classified into small ncRNAs (<200 nt) and long ncRNAs (lncRNAs) (>200 nt).^6^ Most lncRNAs are transcribed by RNA polymerase II. They share common features with the mRNA such as 5′‐capping, splicing, and polyadenylation. The position of the lncRNAs can be in sense or antisense orientation to its neighbouring protein‐coding genes, and within introns or in intergenic regions of the genome. Because of the low binding fidelity property of the RNA polymerase and the low conservation between species, lncRNAs were considered as ‘transcriptional noise’ earlier. LncRNAs have been recently recognized for their crucial functional importance in physiological and diseased conditions.^7^ They have specific expression in different cell types, are localized in different subcellular compartments, and are associated with many diseases.^8^ LncRNAs execute different types of biological regulatory mechanisms—as signals: causing translational modulation of mRNAs following sequence‐specific recognition; as decoys: targeting of chromatin modifiers to DNA through the formation of RNA‐DNA hybrids; as a guide: targeting and sequestration of host factors through RNA secondary structures; and as scaffolds: bringing multiple proteins together in order to form functional ribonucleoprotein complexes.^9^

We have previously reported dys‐regulated lncRNAs in influenza virus‐infected human lung epithelial cells via RNA sequencing analysis and found that PSMB8‐AS1 is a proviral lncRNA that is induced by IFNβ1.^10^ In this study, we identified the new transcript, named as Lnc‐PINK1‐2:5 that is the major transcript in lung epithelial cells. Lnc‐PINK1‐2:5 reduced influenza virus replication via the upregulation of thioredoxin interacting protein (TXNIP).

## 2 MATERIALS AND METHODS

### 2.1 Cell culture

Human alveolar epithelial A549 cell line, HEK cells containing SV40 T‐antigen (HEK293T) and Madin–Darby canine kidney cell line (MDCK) were purchased from American Type Culture Collection (ATCC, Manassas, VA, USA). A549 cells were cultured in F12K media containing 10% foetal bovine serum (FBS) (Atlanta Biologicals, Flowery Branch, GA, USA) and 0.1% penicillin and streptomycin (PS) solution (Life Technologies Corporation, Carlsbad, CA, USA). HEK293T and MDCK cells were cultured in DMEM media containing 10% FBS and 0.1% PS. dCas9‐ KRAB A549 stable cell line was previously generated^10^ and maintained in F12K containing 10% FBS, 0.1% PS and 1 μg/ml of puromycin. Human small airway epithelial cells (HSAECs) were purchased from PromoCell GmbH (Catalog # C‐12642, Sickingenstr, Heidelberg, DEU) and were cultured in SAGM medium (Catalog # CC‐3118, Lonza, Walkersville, MD, USA). Human bronchial epithelial cells (HBECs) were purchased from Lifeline Cell Technology (Catalog # FC‐0035, Frederick, Maryland, USA) and maintained in BronchiaLife™ medium (Lifeline Cell Technology, Catalog # LL‐0023).

### 2.2 Influenza virus

IAV/Puerto Rico/8/34 (PR/8) was purchased from ATCC (Catalog #VR‐95, Manassas, VA, USA). IAV/WSN/1933 (WSN), IAV/Oklahoma/3052/09 (pdm/OK) and A/Oklahoma/309/2006 (H3N2) were kindly provided by Dr. Gillian Air, University of Oklahoma Health Sciences Center. Virus stocks were propagated in specific‐pathogen free eggs (Charles River Laboratories, Houston, TX, USA) as previously described.^10^ The viral stocks were aliquoted and stored in screw cap tubes at −80°C. The titres of the viral stocks were measured by plaque assay.

### 2.3 Virus titre determination

MDCK cells were seeded in 6‐well plates at a density of 5 x 10^5^ cells per well. The next day, cells were washed with sterile Dulbecco's phosphate buffered saline without calcium and magnesium (DPBS) (Lonza, Walkersville, MD, USA) twice. A series of ten‐fold dilutions of virus stock ranging from 10^−3^ to 10^−8^ were prepared in serum‐free medium with 1 μg/ml L‐1‐tosylamide‐2‐phenylethyl chloromethyl ketone‐treated trypsin (TPCK‐trypsin). Cells were incubated with 800 µl of each diluted virus stock for 1 h at 37°C in a 5% CO_2_ incubator and then overlaid with 2x DMEM and heated 2% sea plaque agarose (1:1 ratio) containing 1 μg/ml TPCK‐trypsin. After 72 h, the cells were fixed using 10% neutral buffered formaldehyde and then the overlay was removed. The cells were stained with crystal violet stain solution (Catalog # HT90132, Sigma‐Aldrich, St. Louis, MO, USA) for 2 min and washed. The wells showing countable range of 5–50 plaques were counted and titre (PFU/ml) was calculated using the formula (number of plaques × dilution factor × 1.25).

### 2.4 Virus infection

A549 cells were cultured in 12‐well plates at a density of 0.1 × 10^6^ cells per well with F12K containing 10% FBS and 0.1% PS for 24 h. HBEC cells were plated in 24‐well plate at a density of 8 × 10^4^ cells per well. Cells were washed once with DPBS prior to infection. A549 or HBEC cells were inoculated with IAV at various multiplicity of infections (MOIs) as indicated in serum‐free and PS‐free F12K or BronchiaLife™ medium containing TPCK‐trypsin (0.5 μg/ml), respectively, at 37°C and 5% CO_2_ incubator for 1 h. The virus inoculum was then replaced with serum‐free and PS‐free F12K or BronchiaLife™ medium containing TPCK‐trypsin (0.5 μg/ml). At indicated times post infection, RNAs were extracted using TRI Reagents (Catalog # TR118, Molecular Research Center, Cincinnati, OH, USA) for gene expression by real‐time PCR. Protein samples were collected using M‐PER™ Mammalian Protein Extraction Reagent with Protease and Phosphatase Inhibitor Cocktail (1X) (Catalog # 1861281, Thermo Scientific, Rockford, IL, USA).

### 2.5 RNA sequencing

RNAs were isolated from 3 vector control‐ and 3 Lnc‐PINK1‐2:5‐overexpressing A549 cells infected with PR/8 at an MOI of 0.01 for 48 h. RNA sequencing (RNA_seq) was performed as previously described.^10^ TopHat2 was used to directionally map the paired end reads to the genomic loci of lncRNA (GRCh38 /hg18). Dysregulated mRNAs were identified using cuffdiff analysis based on a fold change of ≥2 and a false discovery rate of <0.05.

### 2.6 Interferon and Poly(I:C) treatment

A549 cells were seeded in 12‐well plates and cultured in F12K medium with 10% FBS and 1% PS overnight. The cells were treated with Poly(I:C) at 100 ng/ml for 24 h or human IFNβ1α (#11415‐1, PBL Assay Science, Piscataway, NJ, USA) at various doses for different times. RNA was extracted for real‐time PCR analysis.

### 2.7 Isolation of cytoplasmic and nuclear RNAs

Cytoplasmic and nuclear RNAs were prepared using a Cytoplasmic and Nuclear RNA Purification Kit (Catalog #21000, Norgen Biotek Corporation, Thorold, ON, Canada) from A549 cells. cDNA was prepared using 500 ng RNA and real‐time PCR was performed. β‐actin (ACTB) and glyceraldehyde 3‐phosphate dehydrogenase (GAPDH) were used as cytoplasmic markers and U2 small nuclear RNA (U2 snRNA) was used as a nuclear marker. The amounts of Lnc‐PINK1‐2 transcripts along with cytoplasmic and nuclear markers in individual fractions were calculated using the formula 2^−ct^ and then multiplied by a dilution factor (total ng of RNA in each fraction/500). The distribution percentage of each gene was calculated as target gene amount in individual fraction divided by the sum of target gene amounts in cytoplasm and nuclei × 100.

### 2.8 Construction of overexpression vector

Lnc‐PINK1:2:5 were PCR‐amplified using GoTaq^®^ DNA Polymerase (Promega, Madison, WI, USA) using human genomic DNAs from A549 cells with following primers: Lnc‐PINK1‐2:5_forward primer: 5′‐ TAACCGCTCGAGGTGCCACAGGGAAGAGAAATGATA ‐ 3′ andLnc‐PINK1‐2:5_reverse primer: 5′ ‐ TAACCGGAATTCCGAGTAGCTGGGATTACAGGTGT ‐ 3′. TXNIP was amplified from the TXNIP Human ORF Clone (Catalog #NM_006472, Origene, Rockville, MD, USA) with the following primers: TXNIP_forward primer: 5′‐ TTTCTCGAGGCCACCAATGGTGATGTTCAAGAAGATCAAGT and TXNIP_reverse primer: 5′‐ TTTGAATTCCTGCACATTGTTGTTGAGGATGC – 3′. The PCR products were inserted into a modified lentiviral vector pLVX (Clontech, Mountain View, CA, USA) the downstream of its green fluorescent protein (GFP) at XhoI and EcoRI as described.^11^

### 2.9 Construction of CRISPR interference vector

Single guide RNA (sgRNA) targeting the promoter region of Lnc‐PINK1‐2:5 was designed using CHOPCHOP (https://chopchop.cbu.uib.no/).^12^ Lnc‐PINK1‐2:5 sgRNA 5′‐ GTGCTGTGGAAAGAAAGGAGGGG ‐ 3′ was cloned into the lentiGuide‐Puro vector (Addgene, Cat# 52963, Watertown, MA, USA) for expressing hU6‐driven sgRNA using BsmBI sites as described in.^13^ 5′ ‐ GGTGGTAGAATAACGTATTAC – 3′ was used as the sgRNA control sequence as previously described.^10^

### 2.10 Construction of shRNA vector

shRNA sequences were designed using BLOCK‐iT™ RNAi Designer and inserted into the hCMV promoter‐driven lentiviral miRZip vector (System Biosciences, Palo Alto, CA, USA) at the downstream of its GFP at BamH1 and EcoR1. The shRNA sequences for TXNIP are forward oligo: 5′‐ GATCCGGATCTGGTGGATGTCAATACTTCAAGAGAGTATTGACATCCACCAGATCCTTTTTG‐3′ and reverse oligo: 5′‐ AATTCAAAAAGGATCTGGTGGATGTCAATACTCTCTTGAAGTATTGACATCCACCAGATCCG −3′.

### 2.11 Lentivirus preparation and titre determination

Lentiviral vector (1.5 µg) was transfected into HEK293T cells along with 6 µg of packaging plasmid psPAX2 (Addgene, Cat#12260) and 3 µg of the envelope plasmid pMD2.G (Addgene, Cat#12259) using 63 μl of 1 µg/µl polyethyleneimine (PEI). After 48 h of transfection, media containing viruses were collected.

To determine the viral titre of a lentivirus containing GFP (lnc‐PINK‐1:2:5 and TXNIP overexpression and TXNIP shRNA), HEK293T cells were seeded at a density of 6 x 10^5^ cells per well in a 12‐well plate and were infected with serial dilutions of lentivirus particles. After 48 h of infection, virus titre was determined by counting GFP‐positive cells (10 fields per well) under a fluorescence microscope.^14^

To determine the viral titre of a lentivirus that does not contain GPF (sgRNA), the titre of a lentivirus was determined using a limiting dilution method.^10^ A549 cells were seeded at a density of 1 × 10^5^/well in 12‐well plates for 24 h and infected with serial dilutions of sgRNA lentivirus for 24 h. The virus was removed after 24 h and fresh media containing puromycin (1 µg/ml) was added to the cells. Cells were cultured in the antibiotic‐containing media for approximately 10 days with a change of medium every 2–3 days until all of the control cells without the lentivirus died. The cells were then stained with crystal violet and live cells colonies were counted with 20× magnification. The lentivirus titre was then calculated by multiplying the number of colonies per well by the dilution factor and expressed as Transforming Units per millilitre (TU/ml).

### 2.12 Real‐time PCR

One µg of the total RNA was reverse‐transcribed into cDNA using Moloney Murine Leukemia Virus (MMLV) reverse transcriptase (Thermo Fischer, Waltham, MA, USA). Primers for the respective genes were designed using Primer3 and listed in Table 1. Real‐time PCR was performed using SYBR Green master mix (Eurogentec, Liege, Belgium) on an ABI 7500 fast system (Applied Biosystems, Foster City, CA, USA). The thermal temperatures were 95°C for 10 min, followed by 40 cycles of 95°C for 15 s, 60°C for 30 s, and 65°C for 30 s. ACTB was used as the endogenous reference gene. The comparative ΔCt method using the equation 2^−ΔCt^ was used to calculate the relative gene expression levels.

**TABLE 1:** List of human and virus primers used for real‐time PCR

Gene name	Forward primer	Reverse primer
Lnc‐PINK1‐2:1	GTGCCACAGGGAAGAGAAATGA	TCAAGCAAGTGGCTTCCT
Lnc‐PINK1‐2:2	TACCTAGAAATTGATCAATATG	CTAACCTTTTCTCTCCAACT
Lnc‐PINK1‐2:3	CAGGAGGCTGAAGCAGGAGA	TTGTTGTTGTCGTCGTTGT
Lnc‐PINK1‐2:4	GACCATCCTGGCTAACATG	CTGGTCTCGAACTCCTGACC
Lnc‐PINK1‐2:5	GAGATCGAGACCATCCTGGCT	CGAGTAGCTGGGATTACAG
ACTB	CATGTACGTTGCTATCCAGGC	CTCCTTAATGTCACGCACGAT
GAPDH	GCACCGTCAAGGCTGAGAAC	TGGTGAAGACGCCAGTGGA
U2snRNA	CATCGCTTCTCGGCCTTTTG	TGGAGGTACTGCAATACCAGG
IFIT1	CAGAACGGCTGCCTAATTTACA	CAGACTATCCTTGACCTGATGATCA
TXNIP	TGTGTGAAGTTACTCGTGTCAAA	GCAGGTACTCCGAAGTCTGT
CLDN2	CGGGACTTCTACTCACCACTG	GGATGATTCCAGCTATCAGGGA
DHRS2	CCTCTGGTAGGGAGCACTCT	CCAGCGCCACTACTGGATTA
HP1BP3	CCATGCCGATTCGTCGAACT	CCTCACTCGAAGTAGCAGGT
NP ‐ PR8	TGTGTATGGACCTGCCGTAGC	CCATCCACACCAGTTGACTCTTG
NS1 ‐ PR8	CGAAATTTCACCATTGCCTT	GTGGAGGTCTCCCATTCTCA

### 2.13 Absolute real‐time PCR quantification

cDNA synthesis using one µg of the total RNA was done as described above. A conventional PCR using GoTaq^®^ DNA Polymerase was performed. The PCR products were purified according to QIAGEN MinElute Gel extraction Kit (Catalog #, 28606 Germantown, MD, USA) and DNA concentrations were measured. Copies/ml of GAPDH and target genes were calculated using the formula; (6.023 × 10^23^ × 10^−6^ × concentration of the purified PCR products (ng/µl))/molecular weight of the PCR product. PCR was performed at the standard curve mode using the serially diluted known template cDNAs (10^0^ to 10^8^) and unknown samples. Absolute copies of target genes were normalized to GAPDH.

### 2.14 Droplet Digital PCR

Absolute Lnc‐PINK1‐2:5 levels in HBEC cells and TXNIP mRNA levels in A549 and HBEC cells were determined by QX200 AutoDG droplet digital PCR (ddPCR) system (Catalog No. #1864100, Bio‐Rad, Hercules, CA, USA) as previously described.^15^

### 2.15 RNA stability determination

A549 cells were cultured in 12‐well plates at a density of 0.4 × 10^6^ cells per well with F12K containing 10% FBS and 0.1% PS for 24 h. The cells were infected with PR/8 at an MOI of 1 for 18 h. Then, cells were treated with serum‐free F12K medium containing actinomycin D (Catalog No. 1229, Tocris, Pittsburgh, PA, USA) at 10 μg/ml for 0, 0.5, 2, 4, 8, 16 h. Cells were collected after respective time points and RNAs were isolated. The expression levels of Lnc‐PINK1‐2:5 were then determined by real‐time PCR.

### 2.16 Inhibitor studies

A549 cells were cultured in 12‐well plates at a density of 0.4 × 10^6^ cells per well with F12K containing 10% FBS and 0.1% PS for 24 h. Cells were washed once with DPBS and inoculated in serum‐free and PS‐free F12K medium containing TPCK‐trypsin (0.5 μg/ml) with PR/8 at an MOI of 1 for 1 h. The inoculum was replaced with serum‐free and PS‐free F12K medium containing TPCK‐trypsin (0.5 μg/ml) with indicated amounts of chemical inhibitors. After 24 h, RNAs were isolated for real‐time PCR. The following chemical inhibitors were purchased from Tocris (Pittsburgh, PA, USA) except BX795, which was obtained from Selleckchem (Houston, TX, USA) and used at indicated concentrations: STAT1 inhibitor, fludarabine (30 µM, Catalog No. 3495); JAK1/2 inhibitor, ruxolitinib (4 µM, Catalog No. 7064); NF‐κb inhibitors, pyrrolidinedithiocarbamate ammonium (PDTC) (30 µM, Catalog No. 0727) and Bay 11–7821 (10 µM, Catalog No. 1744), c‐Myc inhibitor, 10058‐F4 (10 µM, Catalog No. 4406), and TBK1 inhibitor, BX795 (4 µM, Catalog No.S1274).

### 2.17 Western blot

Protein concentration from cell lysates were quantified using Bio‐Rad Protein Assay (Catalog No, 5000006, Bio‐Rad, Hercules, CA, USA). Proteins (10 μg/lane) were separated on 10% SDS‐PAGE and transferred onto nitrocellulose membranes using Trans‐Blot^®^ Turbo™ Transfer System (Bio‐Rad, Hercules, CA, USA). The membranes were blocked for 1 h with 5% non‐fat milk at room temperature, and then, incubated with primary antibodies for overnight in 4°C. After being washed three times with 1× Tris‐buffered saline (pH 7.5) and 0.05% Tween 20, the membranes were incubated with horseradish peroxidase‐conjugated anti‐mouse or rabbit secondary antibodies for 1 h and were again washed three times with Tris‐buffered saline for 5 min. The target proteins were visualized with Super Signal West Pico Chemiluminescent Substrate (Thermo Fischer, Waltham, MA, USA) and analysed with Amersham Imager 600 (GE healthcare system, Pittsburgh, PA, USA). The following antibodies and dilutions were used: mouse anti‐NP, 1:40 dilution (Catalog No, HB‐65, ATCC, Manassas, VA, USA), mouse anti‐NS1, 1:1000 dilution (Catalog No, SC‐130568, Santa‐Cruz biotechnology, Dallas, TX, USA), mouse anti‐β‐actin, 1:3000 dilution (Catalog #, MA5‐15739, Thermo Scientific, Rockford, IL, USA), rabbit anti‐TXNIP, 1;1000 dilution (Catalog #, 14715, Cell Signaling Technology, Danvers, MA, USA), goat anti‐rabbit monoclonal second antibody, 1:1000 dilution (Catalog # 111–035–003, Jackson Immuno Research Laboratories, West Grove, PA, USA), and goat anti‐mouse HRP‐conjugated secondary antibodies 1:2000 (Catalog #115‐0.5–003, Jackson Immuno Research Laboratories, West Grove, PA, USA).

### 2.18 Luciferase reporter assay

A549 cells were seeded at a density of 3 × 10^4^ cells/well in a 96‐well plate and transfected with influenza virus luciferase reporter vector, pHH21‐NP‐3′‐UTR‐LUC‐NP‐5′‐UTR (20 ng), lentiviral Lnc‐PINK1‐2:5 pLVX or vector control (VC) plasmid (100 ng), and a pRL‐TK vector (10 ng) using Lipofectamine 3000 reagent.^16^ Next day, cells were infected with different strains of influenza virus, PR/8 (MOI 0.01), WSN (MOI 0.005), pdm/OK (MOI 0.01), and H3N2 (MOI 0.02) for 48 h. Then, cells were lysed and dual luciferase assay was performed. The results were expressed as the ratio of firefly to *Renilla* luciferase activities.

### 2.19 Immunofluorescence staining

Primary HSAECs were seeded in SAGM complete medium at a density of 8 × 10^4^ cells/well in a 24‐well plate and transduced with Lnc‐PINK1‐2:5 lentivirus at an MOI of 200. After 24 h, the medium was replaced with SAGM medium. On the next day, the cells were infected with PR/8 at an MOI of 0.5 in serum‐free SAGM media containing 0.5 µg/ml of TPCK‐trypsin for 12 h. Cells were then fixed with 100 µl of 4% paraformaldehyde solution at room temperature for 15 min and washed once with DPBS for 5 min. Permeabilization was done with 100 µl of 0.1% of Triton X‐100 at room temperature for 20 min. The cells were washed once with DPBS and incubated with monoclonal mouse anti‐NP, 1:40 dilution (Catalog # HB‐65, ATCC) in DPBS containing 10% of normal goat serum for 1 h at 37°C. Negative control cells were incubated only with DPBS containing 10% of normal goat serum. After 1 h of incubation cells were washed twice with DPBS, then incubated with Alexa fluor 546‐conjugated polyclonal goat anti‐mouse IgG antibodies (1:300, Life technologies, Carlsbad, CA, USA) for 1 h at 37°C, followed by the incubation with 2 µg/ml of Hoechst 3342 (Molecular probes, Waltham, MA, USA) at 37°C for 10 min. After being washed three time with DPBS, cells were observed using a fluorescence microscope. An average of 6 to 8 fields with a total of 400–600 cells were counted, and the percentage of NP‐positive cells were calculated and expressed as the percentage of total cells. Images were captured using Meta Imaging Series 7.7.

### 2.20 Statistical analysis

All experiments were performed for at least three times. Data were presented as the mean ± standard error of the mean (SEM). The statistical significance between two groups were assessed by Student's two‐tailed *t*‐test (unpaired) and comparison of multiple groups were done with analysis of variance (ANOVA), followed by Tukey's post hoc comparison. A *p* value of less than 0.05 was considered as significant.

## 3 RESULTS

### 3.1 Identification and characterization of Lnc‐PINK1‐2:5 transcript

A total of 418 lncRNAs were upregulated and 683 lncRNAs were downregulated in human lung epithelial cells during influenza virus infection based on our previous RNA sequencing analysis.^10^ Lnc‐PINK1‐2 had the high expression in the lungs based on the Noncode database and was chosen for further characterization. According to human genome, GRCh38/hg38 assembly, Lnc‐PINK1‐2 (also named RP5‐930J4.4) is located on chromosome 1, and has four annotated transcripts, Lnc‐PINK1‐2:1 (ENST00000413451), Lnc‐PINK1‐2:2 (ENST00000619933), Lnc‐PINK1‐2:3 (ENST00000616465), and Lnc‐PINK1‐2:4 (ENST00000616189). All of the four transcripts have a size of <1 kb, has two exons and were classified as antisense lncRNAs based on their genomic location of the overlapping protein‐coding gene, heterochromatin protein 1‐binding protein 3 (HP1BP3) on the opposite strand.

**FIGURE 1:**
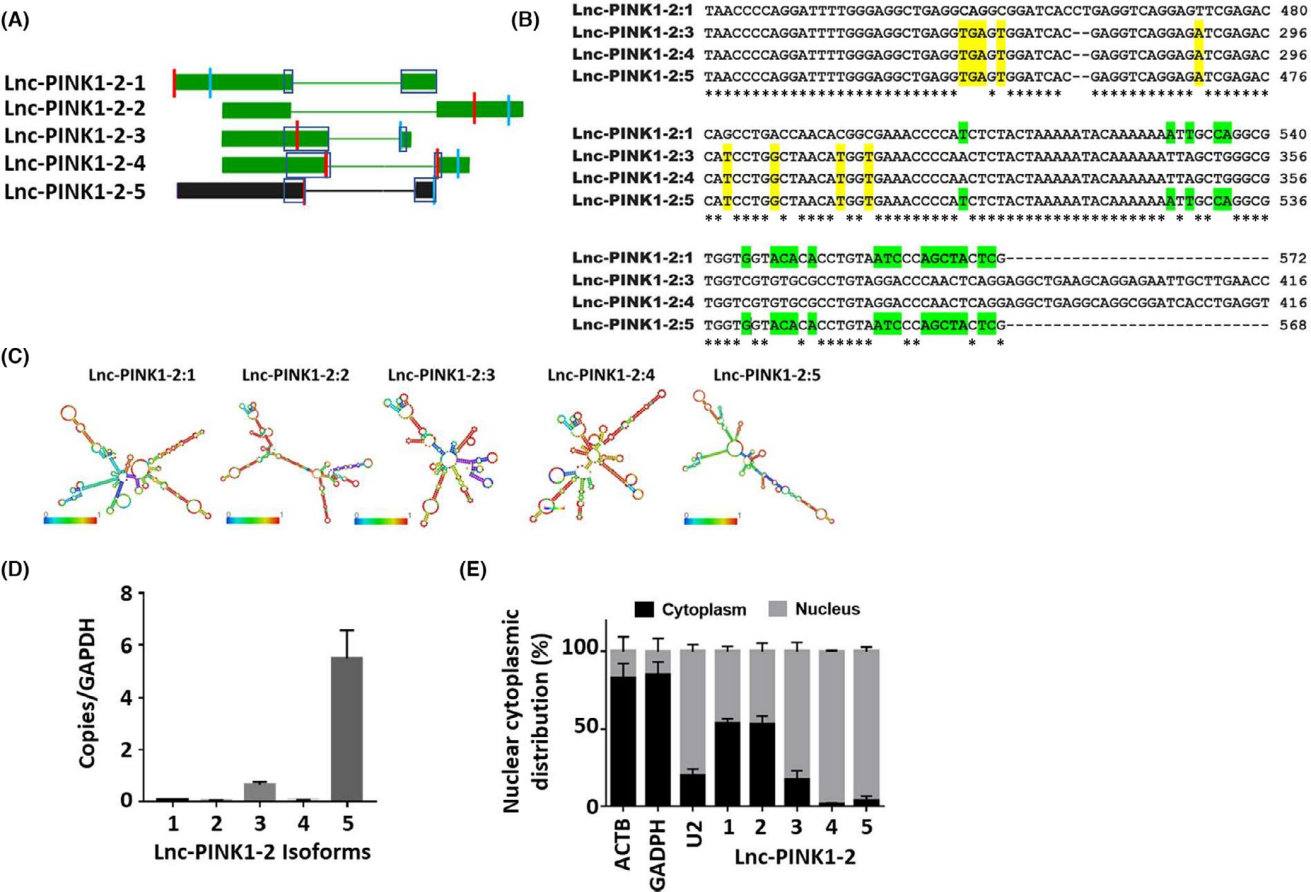
Identification and characterization of Lnc‐PINK1‐2:5. (A) Lnc‐PINK1‐2 has four annotated transcripts (green) according to the Human Dec. 2013 Assembly (GRCh38/hg38) and one new transcript (black) was identified by molecular cloning. The location of the primers used for real‐time PCR for each transcript is indicated in red (forward) and blue (reverse) bars. It is noted that (1) the forward primer for isoform 1 is slightly ahead of isoform 5 in image representation and is distinct from isoform 5, and (2) the forward primer of isoform 4 has sequences from both exons, and the sequence from the exon on the left is not present in isoform 2. Location of sequences from annotated transcripts used for comparison with new transcript as shown in (B) were highlighted in boxes. (B) Sequence comparison of Lnc‐PINK1‐2 transcripts. Due to the differences between isoform 2 and other isoforms, we excluded isoform 2 from the sequence alignment. Identical nucleotides among 4 isoforms were identified by asterisks (_*_) below. Nucleotides highlighted in yellow indicate exact match between transcript 5 and transcripts 3/4, whereas green indicates exact match between transcript 5 and transcript 1. (C) RNA structures as predicted by the RNAfold web server. (D) Copy numbers of Lnc‐PINK1‐2 transcripts in A549 cells. (E) Subcellular localization of Lnc‐PINK1‐2 transcripts in A549 cells (*n* = 3)

When we attempted to amplify lnc‐PINK1‐2:1 from human lung epithelial cell cDNA, we obtained a new transcript that we named lnc‐PINK1‐2:5 (Figure 1A,B). Sequence comparison revealed that the new isoform was a splicing variant that was highly similar to isoform 1 and shared few distinct sequence regions from base pairs 265–314 found in isoforms 3 and 4 but very distinct from isoform 2. We further predicted the secondary structure of Lnc‐PINK1‐2 using the RNAfold web server (http://rna.tbi.univie.ac.at/cgi‐bin/RNAWebSuite/RNAfold.cgi) based on the minimal free energy model. Lnc‐PINK1‐2:5 has a less and shorter branched loops and is much different from other isoforms even though Lnc‐PINK1‐2:5 and LncPINK1:2:1 share highly nucleotide sequence similarity (Figure 1C).

Using absolute PCR, we determined the expression levels of all the lnc‐PINK1‐2 transcripts. Lnc‐PINK1‐2:5 had the highest expression in the lung epithelial cells (Figure 1D). Since the subcellular localization of lncRNAs provide insights on their functional role, we determined the subcellular localization of the Lnc‐PINK1‐2 transcripts. ACTB and GADPH were used as the reference genes for cytoplasmic localization, whereas U2 snRNA was used as a positive control for the nuclear localization. Lnc‐PINK1‐2 isoform 1 and 2 were present in both cytoplasm and nuclei, whereas Lnc‐PINK1‐2 isoform 3, 4, and 5 were mainly localized in nuclei (Figure 1E).

**FIGURE 2:**
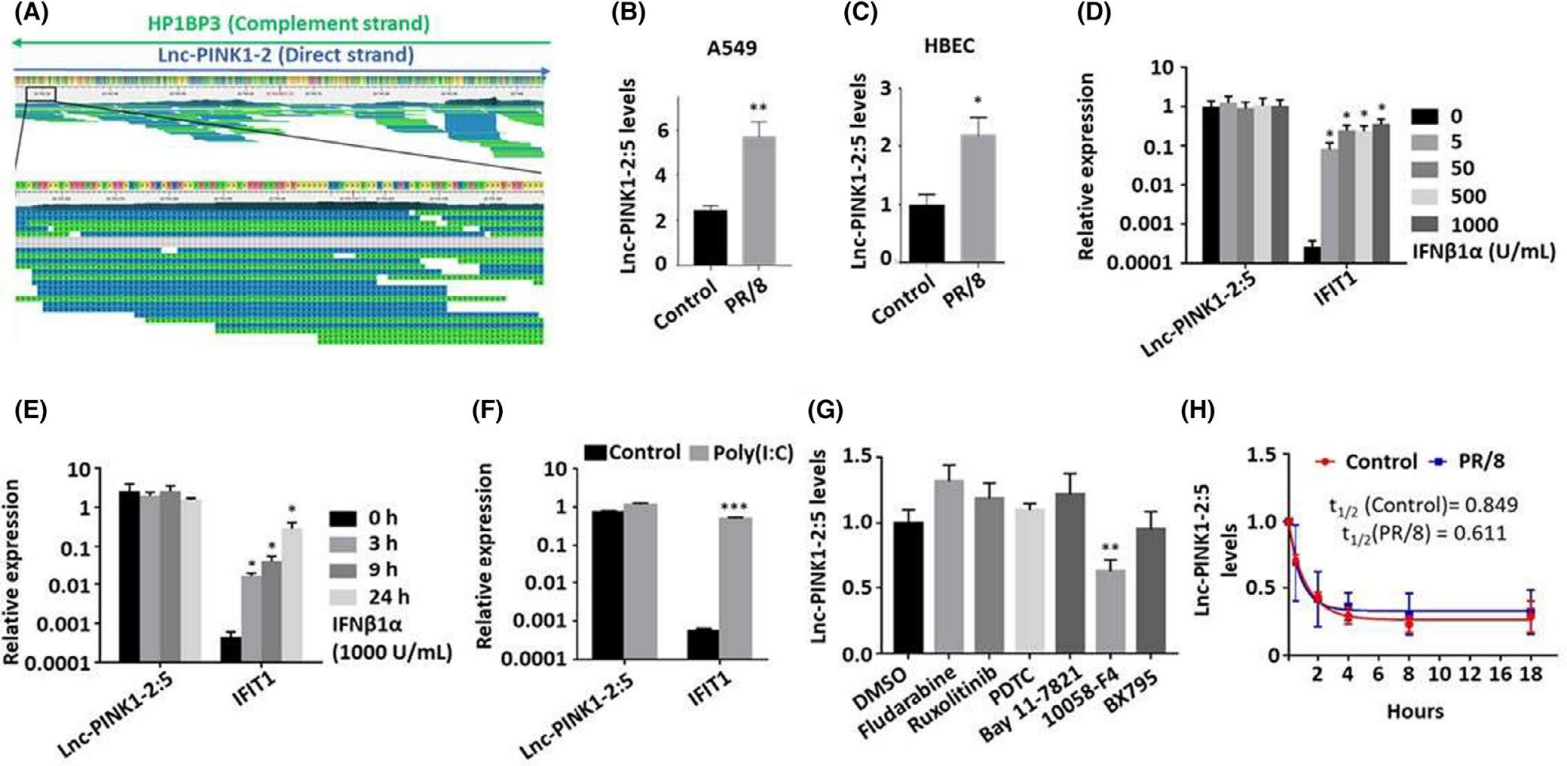
Regulation of Lnc‐PINK1‐2:5 during influenza A virus infection. (A) Strand‐specific genome browser screen shot of the lnc‐PINK1‐2 locus from the RNA‐seq data of one of influenza‐infected cells. Blue colour represents lnc‐PINK1‐2 reads and green colour represents HP1BP3 reads. (B) A549 cells were infected with PR/8 at a MOI of 2 for 24 h. (C) HBEC cells were infected with PR/8 at a MOI of 1 for 24 h. (D, E) A549 cells were treated with IFNβ1α at indicated doses (U/ml) for 24 h or 1000 U/ml for indicated times. (E) A549 cells were treated with Poly(I:C) at 100 ng/ml for 24 h. (F) A549 cells were infected with PR/8 at a MOI of 1 for 1 h and treated with 0.5% DMSO (control), STAT1 inhibitor fludarabine 30 µM, JAK1/2 inhibitor ruxolitinib 4 µM, NF‐κb inhibitors pyrrolidinedithiocarbamate ammonium (PDTC) 30 µM, and Bay 11–7821 10 µM, c‐Myc inhibitor 10058‐F4 10 µM, and TBK1 inhibitor, BX795 4 µM for 24 h. (G) A549 cells were inoculated with PR/8 at an MOI of 1 for 1 h, followed by the treatment with actinomycin (10 μg/ml) for various times. Lnc‐PINK1‐2:5 levels were determined by real‐time PCR (B, D‐H) and ddPCR (C), normalized to β‐actin and were expressed as means ± SE (*n* = 3). B, C. *p < 0.05, **p < 0.01, vs. control, Student's *t*‐test; D‐F, D. *p < 0.05 vs IFIT1 0 h, 0 IFNβ1α, or IFIT1 control, two‐way ANOVA followed by Tukey's comparison; and G. **p < 0.01 vs. DMSO, one‐way ANOVA, followed by Tukey's comparison

Since Lnc‐PINK1‐2:5 is the major transcript in the lung epithelial cells, we chose it for further investigation. By examining strand‐specific genome browser screen shot of the locus from the RNA‐seq data, we can see a clear separation of lnc‐PINK1‐2:5 from the opposite strand of the coding HP1BP3 gene (Figure 2A). Lnc‐PINK1‐2:5 was upregulated in lung epithelial A549 cells and primary human bronchial/tracheal epithelial cells (HBEC) by PR/8 (Figure 2B,C).

Influenza virus activates type I IFN signalling. To examine whether type I IFN affects Lnc‐PINK1‐2:5 expression, we treated A549 cells with IFNβ1α at various doses and different times and determined the expression levels of Lnc‐PINK1‐2:5 transcript. IFIT1, a known IFN‐stimulated gene, was induced by IFNβ1α (Figure 2D,E). However, there was no apparent induction in the expression levels of Lnc‐PINK1‐2:5 transcript. Similar results were observed by using Poly (I:C), a synthetic analogue of double‐stranded RNA that activates toll‐like receptor 3 (Figure 2F) These results suggest that Lnc‐PINK1‐2:5 is not regulated by type I IFN.

We next used the inhibitors for various signalling pathways to determine whether any of the signalling pathways are involved in IAV‐induced upregulation of Lnc‐PINK1‐2:5. A549 cells were inoculated with PR/8 for 1 h, and then, incubated with various inhibitors for 24 h. STAT1 inhibitor fludarabine, JAK1/2 inhibitor ruxolitinib, NF‐κb inhibitors pyrrolidinedithiocarbamate ammonium and Bay 11–7821, and TBK1 inihibitor BX795 had no effects on IAV‐induced Lnc‐PINK1‐2:5 expression (Figure 2G). The results are consistent with the lack of IFNβ1α effects on Lnc‐PINK1‐2:5 expression as these signalling pathways are involved in the type I IFN signalling. Since several lncRNAs were previously reported to be regulated through c‐Myc and influenza virus infection alters c‐Myc mediated metabolic pathways,^17^, ^18^ we used c‐Myc inhibitor to determine its effect on IAV‐mediated Lnc‐PINK1‐2:5 induction. The c‐Myc inhibitor 10058‐F4 reduced IAV‐induced Lnc‐PINK1‐2:5 expression by 38 ± 0.09%, suggesting that IAV‐mediated increase in Lnc‐PINK1‐2:5 expression may be regulated by c‐Myc signalling.

Since gene expression can also be regulated at post‐transcriptional level, we determined the stability of Lnc‐PINK1‐2:5 in control and influenza virus‐infected cells. Lnc‐PINK1‐2:5 expression levels were determined in A549 cells treated with actinomycin D for various times to block transcription. The half‐lives of Lnc‐PINK1‐2:5 transcript in control and PR/8‐infected cells were 0.849 h and 0.611 h, respectively (Figure 2H). These results indicate that influenza virus do not influence the stability of Lnc‐PINK1‐2:5.

### 3.2 Lnc‐PINK1‐2:5 inhibits influenza virus replication

**FIGURE 3:**
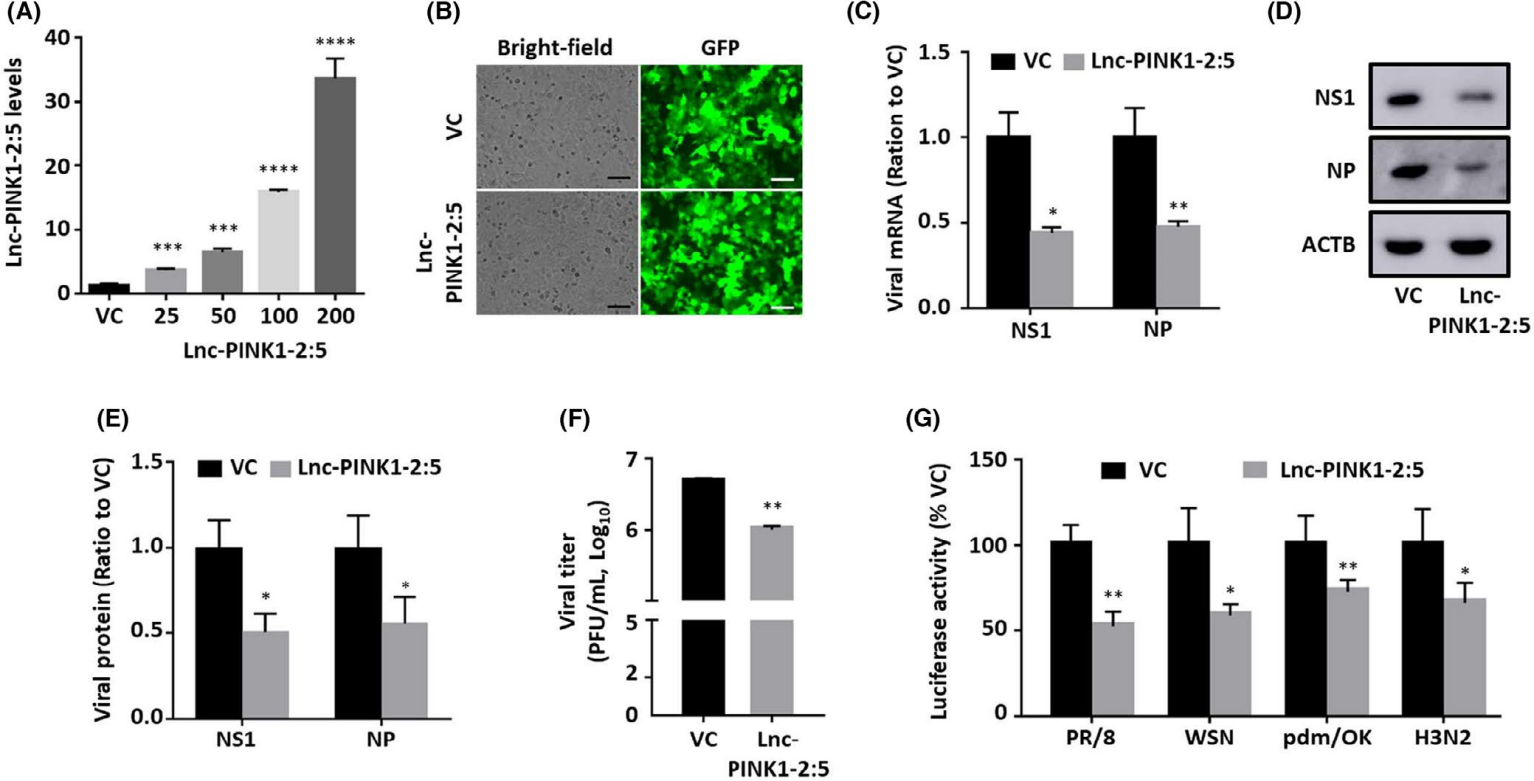
Effect of Lnc‐PINK1‐2:5 on influenza virus replication. (A) A549 cells were transduced with Lnc‐PINK1‐2:5 lentivirus at an MOI of 25–200 or vector control (VC) lentivirus at an MOI of 200 for 48 h. (B‐F) A549 cells were transduced with Lnc‐PINK1‐2:5 or VC lentivirus at an MOI of 200 (B) for 48 h, followed by infection with PR/8 at an MOI of 0.01 for 48 h (C‐F). Lnc‐PINK1‐2:5 (A) and viral mRNAs (C) were determined by real‐time PCR and viral proteins (D) were determined by Western blot. RNA and protein levels were normalized to β‐actin. Viral titre was determined by plaque assay. (G) A549 cells were transfected with 20 ng of influenza virus luciferase reporter vector, 100 ng lentiviral Lnc‐PINK1‐2 or control vector, and 10 ng of pRL‐TK, and infected with PR/8, WSN, pdm/OK, H3N2 strains at various MOI as indicated (*n* = 4). Firefly luciferase activity was normalized to pRL‐TK Renilla luciferase activity in A549 cells. The results are expressed as a ratio to VC‐transfected cells. Antiviral activity of Lnc‐PINK1‐2:5 was depicted as the percentage of reduced luciferase reporter activity. Data was shown as means ± SE. *n* = 3 independent experiments. *p < 0.05, **p < 0.01, and ***p < 0.001 vs. VC. One‐way ANOVA followed by Tukey's comparison for A, two‐way ANOVA followed by Tukey's comparison for C, E, and G. Student's *t*‐test for F

The effects of Lnc‐PINK1‐2:5 on influenza virus replication were determined by overexpression using a lentiviral vector. Infection of A549 cells with Lnc‐PINK1‐2:5 lentivirus resulted in a dose‐dependent increase in Lnc‐PINK1‐2:5 expression compared with vector control‐transduced cells (Figure 3A). An MOI of 200 was chosen for the functional studies because the Lnc‐PINK1‐2:5 expression was the highest. There was not any apparent loss in cell viability by gross microscopic examination at this dose, and GFP images indicated the high transduction efficiency (Figure 3B). Lnc‐PINK1‐2:5‐overexpressing A549 cells were then infected with PR/8 at an MOI of 0.01 for 48 h and viral mRNA and proteins as well as titres were determined. Lnc‐PINK1‐2:5 significantly reduced the mRNA and protein levels of NS1 and NP by approximately 50% (Figure 3C–E). We also observed a noticeable reduction in the viral titre in the cell culture medium of Lnc‐PINK1‐2:5‐overexpressing cells as determined by plaque assay (Figure 3F). These results clearly indicate that Lnc‐PINK1‐2 reduces influenza virus replication.

In order to determine the effects of Lnc‐PINK1‐2:5 on infection with various strains of influenza virus, we performed an IAV luciferase reporter assay. The reporter vector contains a firefly luciferase flanked with the 5′‐ and 3′‐untranslated regions of WSN NP, through which the level of virus replication in the cells at the transcriptional level can be measured. A549 cells transfected with the reporter were infected with PR/8, WSN, pdm/OK and H3N2 at an optimized MOI of 0.02, 0.01, 0.1, and 0.02, respectively, for 48 h. PR/8 and WSN are both laboratory‐adapted strains; pdm/OK is a 2009 Oklahoma pandemic strain and H3N2 is a 2006 human clinic isolate from Oklahoma.^19^, ^20^, ^21^ Overexpression of Lnc‐PINK1‐2:5 reduced approximately 46.35 ± 0.88% of the reporter activity induced by PR/8 (Figure 3G), which was similar to the result from measuring viral mRNA levels (Figure 3C). Lnc‐PINK1‐2:5 also decreased the reporter activity induced by WSN, pdm/OK, and H3N2 by 41.69 ± 0.77%, 33.67 ± 1%, and 31.55 ± 1.04%, respectively, indicating that Lnc‐PINK1‐2:5 has a strain‐independent effect against influenza virus replication.

**FIGURE 4:**
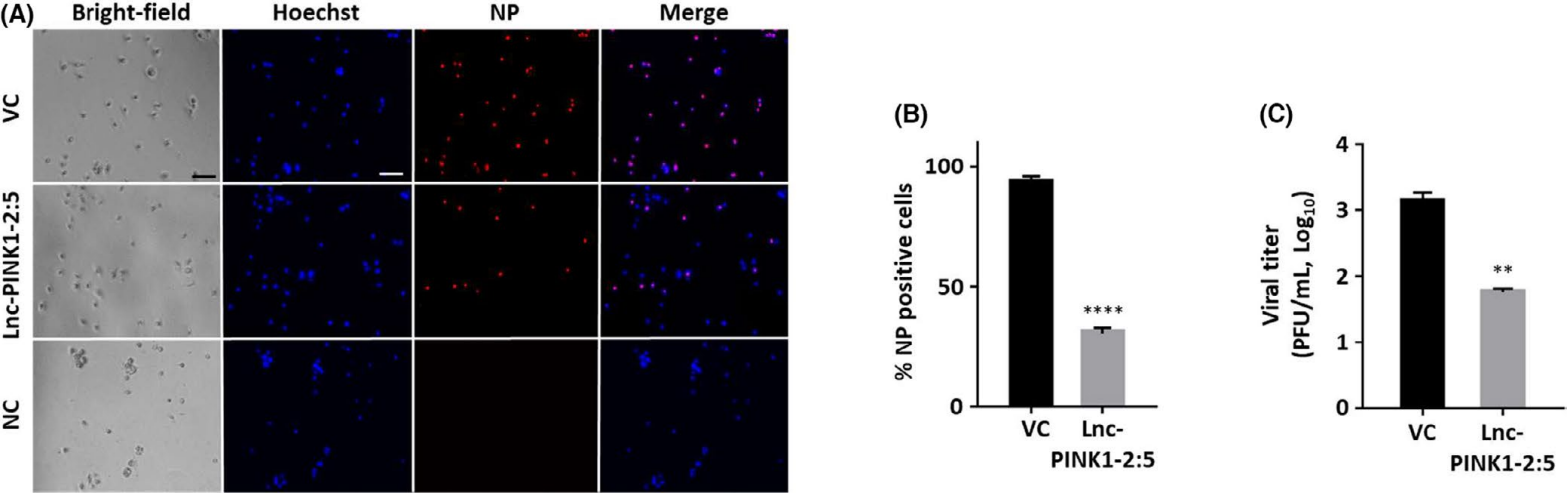
Lnc‐PINK1‐2 inhibits influenza replication in primary cells. Human small airway epithelial cells were transduced with Lnc‐PINK1‐2:5 or vector control (VC) lentivirus at a MOI 200 for 48 h and then infected with PR/8/ at a MOI of 0.5 for 12 h. (A) NP immunofluorescence staining images. Negative controls (NC) were the untransduced cells without primary NP antibody. (B) Quantification of NP‐positive cells. (C) Viral titres in culture media as determined by plaque assay. Data was shown as means ± SE. *n* = 3 independent experiments. **p < 0.01, and ****p < 0.0001 vs. VC (Student's *t*‐test)

To determine whether the effect of Lnc‐PINK1‐2:5 on influenza virus infection can be observed in human primary cells, Lnc‐PINK1‐2:5 overexpressing human small airway epithelial cells were infected with PR/8 at a MOI of 0.5 for 12 h and immunostained for viral NP protein. Negative controls without primary antibodies did not show signals, indicating the specificity of NP antibody (Figure 4A). While approximately 90% of the vector control cells were NP‐positive, only 30% of the Lnc‐PINK1‐2:5 overexpressing cells had NP‐staining (Figure 4B). Compared with vector control, Lnc‐PINK1‐2:5 also reduced viral titre (Figure 4C).

### 3.3 Lnc‐PINK1‐2:5 knockdown enhances influenza virus replication

**FIGURE 5:**
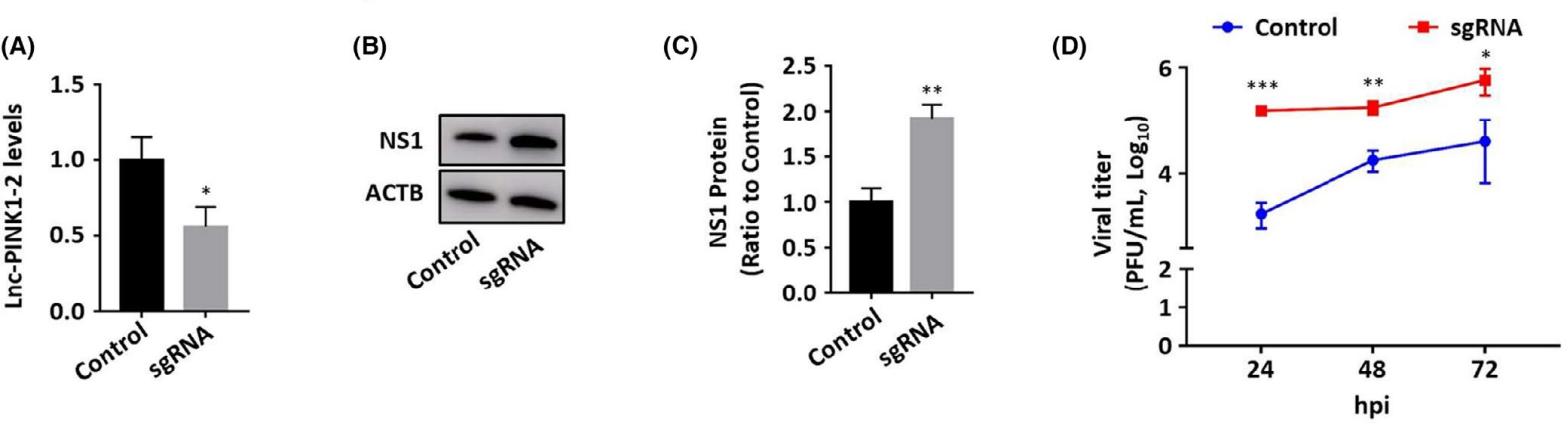
CRISPR interference knockdown of Lnc‐PINK1‐2:5 promotes influenza virus replication. Stable dCAS9‐KRAB A549 cells were transduced with a lentivirus carrying a sgRNA targeting Lnc‐PINK1‐2:5, or its control, and then infected with 2009 pandemic H1N1 OK/09 at MOI 0.05 for 48 h (A‐B) and 24–72 h post infection (hpi) (D). Lnc‐PINK1‐2:5 levels were determined by real‐time PCR (A) and NS1 viral protein levels were determined by Western blot (B). RNA and protein levels were normalized to β‐actin. (D)Viral titre in culture media were determined by plaque assay. Data was expressed as means ± SE. *p < 0.05, **p < 0.01 (*n* = 3). Student's *t*‐test for A, and C and two‐way ANOVA, followed by Tukey's comparison for D

To further confirm the antiviral activity of Lnc‐PINK1‐2:5, we used CRISPR interference to knockdown the endogenous Lnc‐PINK1‐2:5 expression. We designed sgRNAs targeting the promoter region of Lnc‐PINK1‐2:5. We transduced A549 cells stably expressing dCas9‐KRAB with a lentiviral sgRNA or its control and selected with puromycin. Lnc‐PINK1‐2:5 expression levels were significantly reduced by the sgRNA (Figure 5A). The reduction of Lnc‐PINK1‐2:5 increased NS1 protein level of the clinical isolate of 2009 H1N1 pandemic strain, pdm/OK (Figure 5B,C). Knockdown of Lnc‐PINK1‐2:5 also increased viral titre in culture media in a time‐dependent manner (Figure 5D).

### 3.4 Lnc‐PINK1‐2:5 reduces influenza virus replication by upregulating TXNIP

**FIGURE 6:**
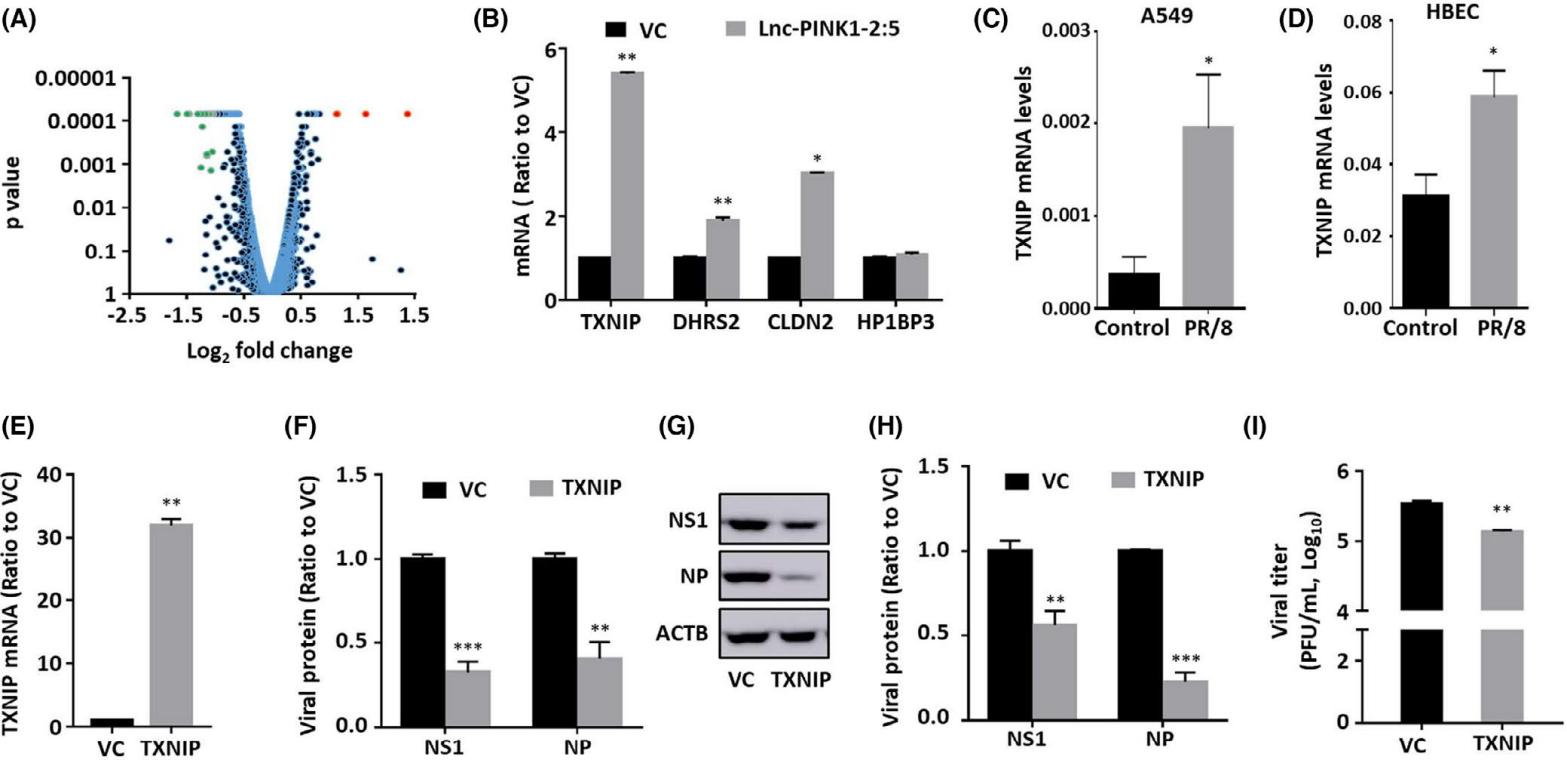
Lnc‐PINK1‐2 upregulates TXNIP gene expression. (A) Volcano plot of differentially expressed mRNAs from the RNA_seq analysis of Lnc‐PINK1‐2:5 and VC overexpressed A549 cells infected with PR/8 at a MOI 0.01 for 48 h. (B) Validation of the upregulated mRNAs performed on the same samples as for RNA‐seq using real‐time PCR. (C, D) TXNIP mRNA levels in A549 cells infected with PR/8 at an MOI of 2 for 24 h or HBEC cells infected with PR/8 at an MOI 1 for 24 h. (E‐I) A549 cells were transduced with a TXNIP or vector control (VC) lentivirus at an MOI of 200, followed by infection with a PR/8 at an MOI of 0.01 for 48 h. mRNA levels were determined by real‐time PCR (B, E, F) or ddPCR (C, D) and viral proteins were determined by Western blot. mRNA and protein levels were normalized to β‐actin and expressed as a ratio to VC for B, E, F, and H. Viral titre was determined by plaque assay. Data was shown as means ± SE. *n* = 3 independent experiments except C, D in which *n* = 4. *p < 0.05, **p < 0.01, and ***p < 0.001 vs. control or VC. Student's *t*‐test for C, D, E, and I. Two‐way ANOVA, followed by Tukey's comparison for B, F, and H

In order to understand the mechanisms of lnc‐PINK1‐2:5 action, we performed RNA‐seq analysis to identify the genes changed in the lnc‐PINK1‐2:5 overexpressing cells. A549 cells were transduced with a lentivirus expressing lnc‐PINK1‐2:5 at a MOI of 200 for 48 h. The cells were then infected with PR/8 at a MOI of 0.01 for 48 h. RNA‐seq was done on the RNA samples from three vector control and three lnc‐PINK1‐2:5‐overexpressing cells. Using a fold change of ≥2 and a false discovery rate of <0.05, we identified 4 upregulated and 19 downregulated genes (Figure 6A and Table 2). Since only 4 genes were upregulated by Lnc‐PINK1‐2:5 during influenza virus infection, we chose the upregulated genes for validation by real‐time PCR. Upregulation of three of the four upregulated genes: TXNIP, claudin‐2 (CLDN2), and dehydrogenase reductase 2 (DHRS2), but not HP1BP3 was validated using real‐time PCR (Figure 6B).

**TABLE 2:** List of the dysregulated genes in lnc‐PINK1‐2:5 overexpressing cells infected with PR/8

Gene symbol	Gene name	FPKM value		Fold change	Differential regulation
		Vector control	Lnc‐PINK1‐2:5		
HP1BP3	Heterochromatin Protein 1 Binding Protein 3	5.54	27.88	5.03	Up
TXNIP	Thioredoxin Interacting Protein	1.03	3.17	3.08	Up
CLDN2	Claudin−2	26.02	57.46	2.21	Up
DHRS2	Dehydrogenase/Reductase 2	0.56	1.23	2.20	Up
BIRC3	Baculoviral IAP Repeat Containing 3	7.73	3.97	0.51	Down
HERPUD1	Homocysteine Inducible ER Protein With Ubiquitin Like Domain 1	8.63	4.41	0.51	Down
IFNL3	Interferon Lambda 3	2.90	1.47	0.51	Down
C11orf96	Chromosome 11 Open Reading Frame 96	1.95	0.97	0.50	Down
HIST1H3B	Histone Cluster 1 H3 Family Member B	30.44	15.15	0.50	Down
HIST1H2BE	Histone Cluster 1 H2B Family Member E	1.94	0.96	0.49	Down
MAFF	MAF BZIP Transcription Factor F	4.30	2.12	0.49	Down
HIST2H2BF	Histone Cluster 2 H2B Family Member F	1.49	0.73	0.49	Down
HIST1H1D	Histone Cluster 1 H1 Family Member D	6.65	3.17	0.48	Down
IFNL2	Interferon Lambda 2	2.59	1.23	0.47	Down
ATF3	Activating Transcription Factor 3	6.63	3.13	0.47	Down
TRIML2	Tripartite Motif Family Like 2	2.91	1.36	0.47	Down
HIST1H2BO	Histone Cluster 1 H2B Family Member O	20.92	9.41	0.45	Down
EGR1	Early growth response protein 1	10.89	4.87	0.45	Down
OR2B6	Olfactory Receptor Family 2 Subfamily B Member 6	3.63	1.62	0.44	Down
HIST2H3D	Histone Cluster 2 H3 Family Member D	16.39	6.97	0.43	Down
IFNB1	Interferon Beta 1	6.11	2.35	0.38	Down
BHLHA15	Basic Helix‐Loop‐Helix Family Member A15	4.05	1.53	0.38	Down
CH25H	Cholesterol 25‐hydroxylase	2.40	0.80	0.34	Down

TXNIP is located on the same chromosome as lnc‐PINK1‐2:5 and was the most upregulated. Additionally, TXNIP has been shown to be a critical component in activating inflammasomes,^22^, ^23^, ^24^ which are linked to influenza virus infection.^25^, ^26^, ^27^ Thus, we selected TXNIP for further studies. TXNIP mRNA levels were increased after influenza virus infection in A549 cells and primary HBEC cells (Figure 6C,D). To determine the role of TXNIP in influenza virus replication, TXNIP was overexpressed in A549 cells using a lentivirus expressing TXNIP, and then, infected with PR/8 at a MOI of 0.01 for 48 h. The TXNIP level was increased by 32‐fold in TXNIP‐overexpressing cells, resulting in 67 ± 0.6% and 59 ± 0.9% reduction in viral NS1 and NP mRNA levels (Figure 6E,F). Overexpression of TXNIP also reduced viral NS1 and NP protein levels by 46 ± 2% and 77 ± 1%, respectively, as well as viral titre in culture media by 0.4 log fold (Figure 6G–I).

**FIGURE 7:**
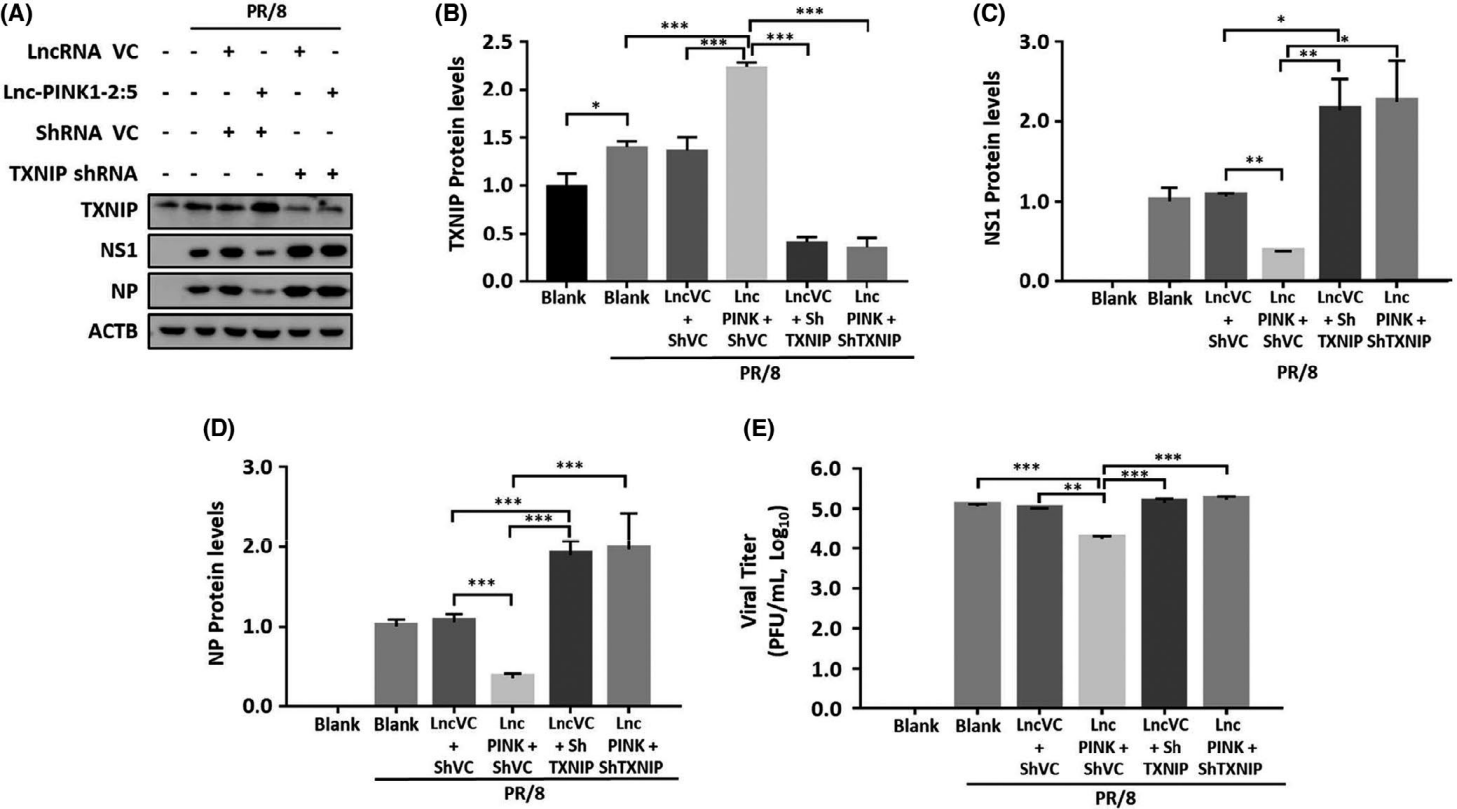
Knockdown of TXNIP abolishes Lnc‐PINK1‐2:5 effect. A549 cells were transduced with lentivirus expressing TXNIP shRNA or its vector control (VC), and/or Lnc‐PINK1‐2:5 or its VC at an MOI of 100. When two viruses were involved, MOI of 50 were used for each virus. The cells were then infected with a PR/8 at an MOI of 0.01 for 48 h. TXNIP and viral proteins were determined by western blot and quantified. mRNA and protein levels were normalized to β‐actin and expressed as a ratio to condition 1 (blank) or 2 (blank with PR/8). Viral titres were determined by plaque assay. Data was shown as means ± SE. *n* = 3 independent experiments. *p < 0.05, **p < 0.001, ***p < 0.0001 (One‐way ANOVA followed by Tukey's comparison)

To examine whether lnc‐PINK1‐2:5‐mediated reduction of influenza virus infection is via TXNIP, we knocked down TXNIP in the vector control‐ and lnc‐PINK1‐2:5‐overexpressing cells to see whether a decrease in TXNIP protein level can reverse lnc‐PINK1‐2:5‐mediated effect. A549 cells were transduced with a lentivirus expressing lnc‐PINK1‐2:5 and/or TXNIP shRNA at a MOI of 100 for 48 h and then infected with PR/8 at a MOI of 0.01 for 48 h. Influenza virus increased TXNIP protein expression by 40 ± 1% relative to the control. Compared to the vector control, TXNIP shRNA reduced TXNIP protein levels by 70 ± 2 and 72 ± 3% without or with lnc‐PINK1‐2:5 overexpression (Figure 7A,B). The reduction in TXNIP protein level increased viral NS1 and NP levels. Lnc‐PINK1‐2:5 overexpression reduced viral NP protein expression and knock‐down of TXNIP abolished the effect (Figure 7A,C,D). Similar results were observed with viral titres (Figure 7E). The result suggests that lnc‐PINK1‐2:5‐mediated antiviral activities are via TXNIP.

## 4 DISCUSSION

LncRNAs have been increasingly recognized to play a crucial role during influenza infection.^28^ In this study, we identified a new transcript, Lnc‐PINK1‐2:5 that was upregulated by influenza virus. We demonstrated that Lnc‐PINK1‐2:5 limited IAV replication through the upregulation of TXNIP.

Lnc‐PINK1‐2 is an antisense lncRNA present in the opposite strand of HP1BP3. To our knowledge, no studies have been reported on this lncRNA so far. According to GRCh38/hg38 assembly, Lnc‐PINK1‐2 has four annotated isoforms. During the process of cloning Lnc‐PINK1‐2 from human lung epithelial cells for functional studies, we identified a new isoform, Lnc‐PINK1‐2:5, which was specifically expressed in human lung epithelial cells among all of the 5 transcripts. This new isoform shares some common sequences with isoform 1, 3, and 4. Secondary structure prediction shows that Lnc‐PINK1‐2:5 is distinct from other 4 isoforms. Lnc‐PINK1‐2:5 was predominantly present in the nucleus, suggesting that it may function in the nucleus.

Most often, the regulation of lncRNAs by influenza virus is via type I IFN signalling. However, a few lncRNAs are regulated through type I IFN‐independent pathway. LncRNA‐155 is regulated by RIG‐I and TLR3, which are critical sensors for type I IFN signalling during viral infection.^29^ Inhibitor studies using ruxolitinib, a JAK1/2 inhibitor, have revealed that BISPR is a STAT‐dependent lncRNA.^30^ Similarly, TSPOAP1‐AS1 has been shown to be regulated by NF‐κB signalling using the NF‐κB inhibitor, Bay 11–7082.^31^ Similar to their sense antiviral genes, ISG20 and MxA, Lnc‐ISG20, and Lnc‐MxA are IFN‐stimulated genes.^32^, ^33^ VIN and LncRNA ACOD1 are induced by influenza virus, but not through IFN signalling.^34^, ^35^

Our current studies showed that Lnc‐PINK1‐2:5 expression was induced by influenza virus, but not IFNβ1α, which is supported by the observations that JAK1/2 and STAT1 inhibitors had no effects on IAV‐induced Lnc‐PINK1‐2:5 expression. Furthermore, NF‐κB inhibitor also did not reduce IAV‐induced Lnc‐PINK1‐2:5 expression. However, we found that c‐Myc inhibitor inhibited IAV‐induced Lnc‐PINK1‐2 expression. These results suggest that Lnc‐PINK1‐2:5 expression is regulated by c‐Myc, but not type I IFN signalling.

Most lncRNAs that have been studied so far regulate influenza virus replication through the modulation of host immune responses.^28^ LncRNA NRAV promotes IAV infection by negatively regulating the expression of IFITM3 and MxA through decreasing histone 3 lysine 4 trimethylation and increasing histone 3 lysine 27 trimethylation at the IFITM3 and MxA transcription start sites.^36^ Lnc‐MxA negatively regulates IFNβ induction by binding to the promoter region of IFNβ upon the formation of RNA:DNA triplexes, and thus, facilitates the replication of influenza virus.^33^ Lnc‐ISG20 competitively binds with miR‐326 and enhances ISG20 protein expression, resulting in an decrease in IAV replication.^32^ IVRPIE exhibits its antiviral activity on influenza viral replication by increasing IFNβ1 and ISGs expression through enrichment of H3K4me3 marks at the transcription start sites of IFNβ1, IRF1, IFIT1, IFIT3, MX1, ISG15, and IFI44 L.^37^ There are few lncRNAs that are known to act by the mechanisms independent of immune response modulation. Two lncRNAs IPAN and PAAN have been found to stabilize the RNA polymerase PB1 and PA, respectively, and promote the viral replication.^38^, ^39^

In this study, we found that Lnc‐PINK1‐2:5 is an antiviral lncRNA because overexpression of Lnc‐PINK1‐2:5 resulted in a decreased viral replication as indicated by the viral RNA, protein and titre levels and knockdown of Lnc‐PINK1‐2:5 had an opposite effect. We also provide evidence that Lnc‐PINK1‐2:5 exerts its antiviral activity via the upregulation of TXNIP, which is supported by (1) TXNIP overexpression decreased and TXNIP knockdown increased influenza virus infection, suggesting that TXNIP is an antiviral factor, and (2) knockdown of TXNIP abolished the antiviral activity of Lnc‐PINK1‐2:5.

TXNIP is a redox protein that regulates the functions of pancreatic β‐cells.^40^ It is a negative regulator of thioredoxin, which plays an important protective role against oxidative stress.^41^ TXNIP is identified as an NLRP3‐binding protein, mediating oxidative stress and ER stress‐mediated activation of NLRP3 inflammasomes.^22^, ^23^, ^24^

In an influenza virus polymerase interaction network study using the yeast two‐hybrid system, TXNIP was found to interact with the viral polymerase protein PB2.^42^ However, this interaction was not further validated, and whether this interaction influences the polymerase activity remains to be determined.

In conclusion, Lnc‐PINK1‐2:5 is an anti‐influenza lncRNA acting through the upregulation of TXNIP.

## CONFLICT OF INTEREST

The authors have no conflict of interest.

## AUTHOR CONTRIBUTIONS

**Samuel Pushparaj:** Formal analysis (lead); Investigation (lead); Methodology (lead); Validation (lead); Visualization (lead); Writing – original draft (lead). **Zhengyu Zhu:** Formal analysis (equal); Investigation (equal). **Chaoqun Huang:** Data curation (equal); Formal analysis (equal); Investigation (equal); Methodology (equal). **Sunil More:** Formal analysis (equal); Investigation (equal). **Yurong Liang:** Formal analysis (equal); Investigation (equal); Methodology (equal). **Kong Lin:** Formal analysis (equal); Investigation (equal). **Kishore Vaddadi:** Formal analysis (equal); Investigation (equal). **Lin Liu:** Conceptualization (lead); Funding acquisition (lead); Investigation (lead); Project administration (lead); Supervision (lead); Validation (lead); Writing – original draft (lead).

## Data Availability

The data that support the findings of this study are available in GEO database (GSE179747) at NCBI.
